# Recent Research Progress (2015–2021) and Perspectives on the Pharmacological Effects and Mechanisms of Tanshinone IIA

**DOI:** 10.3389/fphar.2021.778847

**Published:** 2021-11-08

**Authors:** Chenhui Zhong, Zuan Lin, Liyuan Ke, Peiying Shi, Shaoguang Li, Liying Huang, Xinhua Lin, Hong Yao

**Affiliations:** ^1^ Department of Pharmaceutical Analysis, School of Pharmacy, Fujian Medical University, Fuzhou, China; ^2^ Department of Traditional Chinese Medicine Resource and Bee Products, College of Animal Sciences (College of Bee Science), Fujian Agriculture and Forestry University, Fuzhou, China; ^3^ Higher Educational Key Laboratory for Nano Biomedical Technology of Fujian Province, Fujian Medical University, Fuzhou, China; ^4^ Fujian Key Laboratory of Drug Target Discovery and Structural and Functional Research, Fujian Medical University, Fuzhou, China

**Keywords:** tanshinone IIA, molecular mechanism, pharmacological effects, pharmacokinetics, bioinformatic analysis

## Abstract

Tanshinone IIA (Tan IIA) is an important characteristic component and active ingredient in *Salvia miltiorrhiza*, and its various aspects of research are constantly being updated to explore its potential application. In this paper, we review the recent progress on pharmacological activities and the therapeutic mechanisms of Tan IIA according to literature during the years 2015–2021. Tan IIA shows multiple pharmacological effects, including anticarcinogenic, cardiovascular, nervous, respiratory, urinary, digestive, and motor systems activities. Tan IIA modulates multi-targets referring to Nrf2, AMPK, GSK-3β, EGFR, CD36, HO-1, NOX4, Beclin-1, TLR4, TNF-α, STAT3, Caspase-3, and bcl-2 proteins and multi-pathways including NF-κB, SIRT1/PGC1α, MAPK, SREBP-2/Pcsk9, Wnt, PI3K/Akt/mTOR pathways, TGF-β/Smad and Hippo/YAP pathways, etc., which directly or indirectly influence disease course. Further, with the reported targets, the potential effects and possible mechanisms of Tan IIA against diseases were predicted by bioinformatic analysis. This paper provides new insights into the therapeutic effects and mechanisms of Tan IIA against diseases.

## Introduction

*S. miltiorrhiza* has been used for thousands of years as a traditional Chinese medicine in Asia. Modern science also has a considerable attention on the study of the various components of *S. miltiorrhiza*. Tanshinone IIA (Tan IIA) is the main fat-soluble component of the dried root of *S. miltiorrhiza*, which is widely used clinically. Sodium Tan IIA sulfonate, a water-soluble derivative of Tan IIA, has been approved by China State Food and Drug Administration (CFDA) for the treatment of cardiovascular diseases. Moreover, studies on the pharmacodynamics and pharmacological mechanism of Tan IIA are ongoing. Recent reviews introduced the role of Tan IIA in various diseases. Fang et al. (2020) mainly introduce the effect of Tan IIA in anti-cancer, Shi et al. (2019) described the effects of Tan IIA in the treatment of liver disease, Guo et al. (2020) summarized the anti-inflammatory and anti-oxidant, anticoagulant, antithrombotic and neuroprotective roles, and related effect mechanisms against cardiovascular disorders (i.e., atherosclerosis, hypertension) alzheimer’s disease and carcinoma progression. A recent review (Ansari et al., 2021) also summarized the effects of Tan IIA in treatment of cardiovascular diseases, cerebrovascular diseases, cancer, diabetes, obesity and neurogenic diseases with a special convergence on nano-based drug delivery formulations. These suggests that Tan IIA is a multi-target and multi-pathway active ingredient, which could have promise potentials in clinical usage. However, up to now, an integrative and systematic pharmacology analysis for Tan IIA’s multi-target and multi-pathway is still lacked, which is undoubtedly unfavorable for understanding the comprehensive therapeutic effects and mechanisms of Tan IIA against diseases.

In this paper, we retrospect the recent progress mainly during 2015–2021 on the investigation of Tan IIA’s pharmacokinetics, pharmacological activities and mechanisms towards cancers and cardiovascular, nervous, respiratory, urinary, digestive and motor systems diseases. Further, based on the reported effect targets and pathways, an integrative and systematic prediction on the possible mechanisms of Tan IIA against diseases was performed through Kyoto Encyclopedia of Genes and Genomes (KEGG) pathway and disease ontology semantic and enrichment (DOSE) analyses. This paper provides comprehensive insights into the therapeutic effects and mechanisms of Tan IIA against diseases, which will undoubtedly promote the development and usage of Tan IIA and the *S. miltiorrhiza* products in the clinic.

## Basic Properties

*miltiorrhiza* is the dry root and rhizome of Salvia miltiorrhiza Bge. The traditional usage of Salvia miltiorrhiza include making decoction, tablet, powder, compatibility with other traditional Chinese medicine, etc. The main active components of *S. miltiorrhiza* can be divided into two parts, water-soluble salvianolic acids and lipid soluble tanshinones. The lipid soluble components mainly include tanshinone I, Tan IIA, dihydrotanshinone I, cryptotanshinone, etc. The water-soluble components include Danshensu, salvianolic acid B, protocatechualdehyde, etc. The major active components of *S. miltiorrhiza* have cardiovascular protective effects and may have synergistic effects with each other (Li ZM. et al., 2018), among which Tan IIA has been widely reported due to its powerful pharmacological activity in the treatment of cardiovascular diseases (Gao et al., 2012; Fang et al., 2018c). Tan IIA (Molecular Formula：C_19_H_18_O_3_, MW：294.3), is a diterpenoid quinone from *S. miltiorrhiza* with red needle crystal. The reported gastrointestinal absorption properties (the *in vitro* bidirectional permeability in Caco-2 cell monolayers from literature) and the predicted physiochemical properties, including *P*_app(basolateral→apical)_ (0.98 × 10^−6^ cm/s), *P*_app(apical→basolateral)_ (11.81 × 10^−6^ cm/s), solubility in water (0.0104 mg/ml) and octanol–water partition coefficient (cLog*P*, 4.16), hint its poor oral bioavailability (Yao et al., 2018).

### Potential Toxicity

The cell viability of H9c2 cells treated with Tan IIA at 0–10 µM for 24 h (Gu et al., 2016) and the apoptosis rate of cells treated with Tan IIA at 50 µM for 2 h in myocardial microvascular endothelial cells (Cui et al., 2016) were not significantly different from those in the normal control group. The *in vitro* potential toxicity was also evaluated by the zebrafish embryo model and found no teratogenic effects when the concentration of Tan IIA was below 5 µM in both the chorionic and dechorionated embryo groups. At high concentration, it exhibited severe growth inhibition, developmental deformity and cardiac toxicity (Wang T. et al., 2017). The practical preparation of Tan IIA is sodium tanshinone IIA sulfonate (STS). There have been many clinical trials. In a clinical trial of the potential cardioprotective effect of STS in patients with non-ST elevation acute coronary syndrome, 192 patients were given STS and 180 were given saline, and the results showed that 30-days major adverse cardiac events occurred in 18.8% of the STS group and 27.2% of the control group, and the incidence of bleeding was similar between patients receiving STS and control group. The STAMP trial indicated that STS decreasing the amount of myocardial injury in patients without any detrimental side effects (Mao et al., 2021).

### Tissue Distribution and Herb-Drug Interactions

Tan IIA is mainly administered orally and intravenously in previous reports. After oral administration, the distribution of Tan IIA in rat tissues was determined by liquid chromatography tandem mass spectrometry (LC/MS/MS). Its tissue concentrations decreased in the order of stomach > small intestine > lung > liver > fat > muscle > kidneys > spleen > heart > plasma > brain > testes. It had a wide tissue distribution but the oral bioavailability was extremely low (Bi et al., 2007). Improving the type of preparation, such as the use of lipid nanocapsules (Ashour et al., 2020) or interactions among different active components of *S. miltiorrhiza* and herb-drug interactions can increase the bioavailability and plasma concentrations of Tan IIA obviously. For example, compared with oral administration of pure Tan IIA, the AUC_0-∞_ of Tan IIA was significantly higher in the prostate, liver, and heart of rats receiving *Salvia miltiorrhiza* extract (Wang D. et al., 2020). Therefore, other ingredients in *Salvia miltiorrhiza* extract may promote the distribution of Tan IIA. After intravenous injection of Xiangdan injection to rats, Jiangxiang can inhibit the metabolism and excretion of diterpenoid quinones then improve the bioavailability of Tan IIA (Shi B. et al., 2020). In addition, other traditional Chinese medicines such as *Panax Notoginseng* saponins extracts and borneol combined with *Salvia miltiorrhiza* enhance Tan IIA and salvianolic acid B transport to the brain, shortened t_max_ of Tan IIA in plasma and brain. (Zhang et al., 2021).

### Metabolism

The main metabolic pathway of Tan IIA is hydroxylation, which is responsible for CYP2A6 in liver microsomes (Liu et al., 2009), Studies have shown that Tan IIA exhibited different modes of inhibitory effects on the metabolism of model probe substrates, referring to CYP2C8, CYP1A2, CYP2C9 (Wang et al., 2010; Xu MJ. et al., 2018). And Tan IIA can activate human PXR and consequently induce the expression of the *CYP3A4* gene. Therefore, it should be cautious to taking drugs metabolized by CYP3A4 when using *S. miltiorrhiza* products (Yu et al., 2009). And the major phase II metabolism pathway of Tan IIA is glucuronidation and the metabolites are excreted via bile (Sun et al., 2007). Wei et al. described that zebrafish could successfully imitate regular phase I metabolism. In combination with using HPLC/IT-MS^n^ analysis, they found two monohydroxy Tan IIA (MW 310) and one dihydroxy Tan IIA (MW 326), suggesting that Tan IIA underwent a metabolic transformation in a zebrafish model, which was extensively similar to that in rats (Wei et al., 2012). In addition, some novel metabolites of Tan IIA were putatively identified by UHPLC-Q-Exactive Orbitrap mass spectrometry, such as methylation, dehydration, decarbonylation,reduction reaction, glucuronidation, and glycine linking products (Liang et al., 2019).

## Anti-Cancer

**FIGURE 1:**
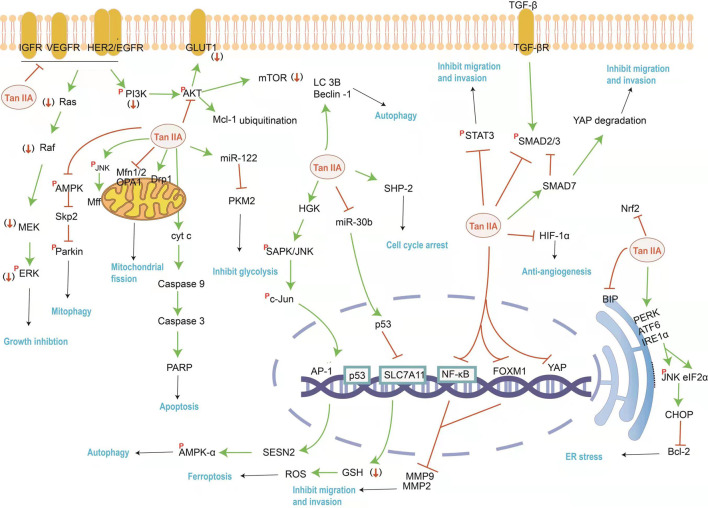
The mainly affected targets and pathways by Tan IIA against cancer. Tan IIA can inhibit cancer cell proliferation and metastasis, induce cell cycle arrest and autophagy, as well as induced ER stress and block energy supply, etc. The orange red symbols (↓), ┤ and *p* represent down-regulation, inhibition and phosphorylation of proteins respectively, and green line-arrow “→” means singal transduction.

Despite medical advances in surgical technology, radiation, chemotherapy and gene targeted therapy, the development of monomeric components from traditional Chinese medicines has always been the focus of cancer treatment research. Tan IIA has a cytotoxic effect on cancer cells, inhibits cancer cell proliferation and activates cancer cell apoptosis by promoting autophagic and inducing cell cycle arrest, and restrains cancer invasion, migration and metastasis (Zhang et al., 2019b). The summarized mechanism of Tan IIA against cancers is shown in Figure 1 and Table 1.

**TABLE 1:** Summary of the anticancer effects and mechanisms of Tan IIA.

Cancers	Animal/Cell	Mechanism	Effects	References
Cervical cancer	SiHa, HeLa and C33a cells mice	↓Akt/mTOR ,HIF-1α	↓cancer cell viability and induced apoptosis	Liu et al. (2019b)
Gastric carcinoma	AGS cells	↓VEGFR and HER2	↑cancer cells apoptosis	Su, (2018b)
		↑ PARP and caspase-3		
Pancreatic cancer	MiaPaCa-2 cells	↓ EGFR, IGFR and VEGFR, Ras/Raf/MEK/ERK and PI3K/AKT/mTOR pathways	↑cancer cell apoptosis	Su, (2018a)
Gastric cancer	AGS cells	↓ EGFR, IGFR, PI3K, AKT and mTOR	↑cancer cell apoptosis	Su and Chiu, (2016)
Non-small cell lung cancer	HCC827, H1975, and A549 cells	↓EGFR-Akt	↓tumor growth	Gao et al. (2020)
	HBE, NL20, MRC5 cells			
	Athymic nude mice			
Ovarian cancer	TOV-21G cells	↑miR-205	↑apoptosis	Li et al. (2018c)
		↓survivin		
Nasopharyngeal carcinoma	Human nasopharyngeal carcinoma cells	↑ PARP, p53, cyclin B1/CDC2 and caspase-3	↓proliferation and induces apoptosis	Liu et al. (2019a)
Hepatocellular carcinoma	HepG2 and Hep3B cells	↑p53, SHP2	↑cell death	Ren et al. (2017)
		↓miR30b		
Gastric cancer	BGC-823 and NCI-H87 cells	↑ p53,ROS	↓cell proliferation	Guan et al. (2020)
		↓SLC7A11,GSH		
Oral squamous cell carcinoma	SCC-9 cells	↑ Beclin-1/Atg7/Atg12-Atg5	↑cell death	Qiu et al. (2018)
	BALB/c-nu mice	↓PI3K/Akt/mTOR signaling		
Glioma	Glioma cells	↓*p*-PI3K and *p*-Akt	↓cell viability	Ding et al. (2017)
		↑LC3B and Beclin-1	↑apoptosis, autophagy	
Osteosarcoma	143B, MG63 cells A549 cells	↑ SESN2/AMPK-α	↑autophagy	Yen et al. (2018)
Oral squamous cell carcinoma	Human oral squamous cell carcinoma SCC090	↑ Beclin-1, Atg5, LC3-II	↑sensitize SCC090 to radiation	Ding et al. (2016)
Prostate cancer	PC-3 cells	↑ Beclin-1, and LC3 II,cleaved caspase-3	↑apoptosis and Autophag	Li et al. (2016a)
Osteosarcoma	MG-63 cells	↑ROS,caspase−3, −8 and −9, and cleaved-PARP	↓cell proliferation	Ma et al. (2016)
colorectal cancer	HCT-116 cells	↓HIF-1α	↓angiogenesis	Zhou et al. (2020)
Nasopharyngeal carcinoma	HNE-1cell	↓ MMP-2 and MMP-9	↓the migration and invasion	Zhou et al. (2018)
		↑p65 and p50		
Cervical cancer	Hela cells, C33 A, and healthy primary normal cervical epithelial cells HcerEpic	↓ YAP transcriptional activity	↓CC stem cells formation, migration and invasion	Qin et al. (2018)
Gastric cancer	SGC-7901 cells	↓MMP-2, MMP-9 and FOXM1	↓SGC-7901 cell proliferation and migration	Yu et al. (2017)
Bladder Cancer	BCa cell lines, 5,637, BFTC and T24	↓STAT3-CCL2 Signaling	↓Epithelial-Mesenchymal Transition	Huang et al. (2017b)
Gastric cancer	SNU-638, MKN1 and AGS cells nude mouse	↓STAT3, Bcl-2	↓cancer cell proliferation	Zhang et al. (2018b)
		↑ Bax and cleaved caspase-3		
Liver cancer	liver cancer tissues	↓ Bcl2, *p*-SMAD2, *p*-SMAD3, and YAP	↓cell proliferation, migration, and invasion	Ma et al. (2019)
	HL-7702,Bel-7404 and SMMC-7721 cells mice	↑SMAD7	↑apoptosis	
Esophageal carcinoma	Eca-109 cells	↑CytC and caspase-9, CHOP	↓Cell viability	Zhang et al. (2017)
		↓BIP	↑apoptosis rate	
Osteosarcoma	NOD-SCID mice implanted with 143B cells	↓ Mfn1/2,Opa1	↑apoptosis	Huang et al. (2017a)
		↑Drp1		
Pancreatic cancer	Immunodeficiency mice BxPC-3 cells	↑PERK, ATF6, caspase-12, IRE1α, eIF2α, *p*-JNK, CHOP and caspase-3	↑ER stress and apoptosis	Chiu and Su, (2017)
		↓Bcl-2		
Colorectal cancer	SW837 and SW480 cells	↑*p*-JNK, Mff	↑apoptotic	Jieensinue et al. (2018)
Osteosarcoma	MG63 cells	↓ AMPK,Nrf2	↓survival, migration, and proliferation	Xie et al. (2020a)
Colorectal cancer	SW837 and SW480 cells	↓AMPK/Skp2/Parkin pathway	↓protective mitophagy	He and Gu, (2018)
			↑mitochondrial apoptosis and cancer cell death	

### Cell Proliferation

PI3K/AKT/mTOR and RAS/RAF/MEK/ERK pathways are the two most common abnormally regulated kinase cascade signaling pathways in tumor progression. Both pathways represent important signal transduction mechanisms that promote the proliferation and survival of cancers driven by growth factor receptors such as factor 1 receptor (IGF1R), vascular endothelial growth factor receptor (VEGFR) and epidermal growth factor receptor (EGFR) or human epidermal growth factor receptor 2 (Her2) (Su, 2018a). Tan IIA induces cell apoptosis by decreasing the expression of EGFR, IGF1R, Her2 and VEGFR in a time and dose-dependent manner and by double blocking the Ras/Raf/MEK/ERK and PI3K/AKT/mTOR pathway *in vitro* and *in vivo* (Su and Chiu, 2016; Su, 2018b). Furthermore, studies have confirmed that Tan IIA can be used as an EGFR inhibitor to reduce the level of myeloid cell leukemia 1 (Mcl-1) protein by ubiquitin, and target EGFR-Akt-Mcl1 axis to inhibit non-small cell lung cancer (NSCLC) (Gao et al., 2020). By down-regulating VEGFR2/Akt pathway, Tan IIA can improve the sensitivity of drug-resistant NSCLC cells to Gefitini (Wang R. et al., 2019). Molecular docking showed that the mechanism of action of Tan IIA as DNA intercalator and topoisomerase II inhibitor was similar to that of its reference drug doxorubicin (Ghanem et al., 2020). Therefore, Tan IIA also can enhance the sensitivity of doxorubicin to drug-resistant gastric cancer cells (Xu Z. et al., 2018). More evidence suggests that noncoding RNAs such as miRNAs and lncRNAs play key roles in many biological processes. Tan IIA may directly upregulates miR-205 and in turn downregulate survivin, and eventually induce ovarian carcinoma TOV-21G cells apoptosis (Li N. et al., 2018).

### Cell Cycle Arrest

It has been proved that upregulation of p53 can lead to cell cycle arrest and the expression of numerous apoptosis-associated genes (Liu B. et al., 2019). Research shows that upstream of p53 is miR30b, and its specific transcription factor is PTPN11. Tan IIA stimulating both PTPN11 and its encoded protein SHP2 induces HepG2 cell death and cell cycle arrest at G1/G0 checkpoints (Ren et al., 2017). p53 upregulation by Tan IIA also causes down-regulated *SLC7A11*, a target gene of p53, increasing GSH consumption then lead to increased intracellular ROS levels, which induce ferroptosis. P53 knockdown and Ferrostatin-1, an inhibitor of lipid peroxidation, attenuated Tan IIA-induced lipid peroxidation and ferroptosis in gastric cancer cells BGC-823 xenograft model (Guan et al., 2020). Combined application of Tan IIA and Andro can promote the crosstalk between ROS and p53, thus promoting cell apoptosis (Li et al., 2020c).

### Autophagy

Tan IIA promotes the autophagy process in a multipronged manner. Tan IIA treatment can trigger the generation of autophagy in a classical Beclin-1-dependent manner and upregulate the expressions of autophagy-associated proteins LC3B and Beclin-1 (Ding et al., 2017). Knockdown of the Beclin-1 blocked the effect of Tan IIA on oral squamous cell carcinoma cell SCC-9 cells both *in vivo* and *in vitro* (Qiu et al., 2018). Autophagy plays an important role in the mechanism of damage repair when treated with a low dose of Tan IIA for a shorter time. While treated with a high dose of Tan IIA for a long time, autophagic cell death contributed to apoptosis (Ma et al., 2016). Tan IIA induced the accumulation of intracellular ROS in human prostate cancer PC-3 cells, which further induced apoptosis and autophagy. The ROS scavenger N-acetyl-L-cysteine (NAC) efficiently inhibited the expression of Beclin-1, LC3-II, and cleaved caspase-3 which were apoptosis and autophagy-associated proteins (Li C. et al., 2016). Therefore, Tan IIA can improve the sensitivity of radiotherapy due to enhanced ROS generation and autophagy (Ding et al., 2016). In addition, Tan IIA-mediated autophagy occurred in a sestrin 2 (SESN2)-dependent manner. SESN2 is DNA damage - and oxidative stress-induced protein and can inhibit mammalian target of rapamycin complex 1 by activating AMP-activated protein kinase (AMPK), while accelerating autophagy (Faubert et al., 2013). The HGK (MAP4K4 or mitogen activated protein kinase kinase)-SAPK/JNK-Jun signal axis can be recruited to the SESN2 promoter to activate SESN2/AMPK-α and induce Tan IIA -mediated autophagy and osteosarcoma growth inhibition (Yen et al., 2018).

### Metastasis

In the process of tumor metastasis, MMPs catalyze the decomposition of the extracellular matrix (Yang et al., 2005), thus enhancing the migration and invasive potential of cancer cells. Tan IIA decreases the expression of MMP-2 and MMP-9 through down-regulation of the NF- kB pathway *in vitro* and *in vivo* (Zhou et al., 2018), and improves the sensitivity of colon cancer cells to 5-FU treatment (Bai et al., 2016). Furthermore. Forkheadboxm 1 (FoxM1) binds to sequence-specific motifs on DNA through its DNA-binding domain and activates proliferation, migration and epithelial–mesenchymal transition (EMT) -associated genes. Overexpression of FoxM1 increased MMP-2 and MMP-9 expression, while knockdown of FoxM1 by siRNA inhibited gastric cancer cell proliferation and migration to the same extent as Tan IIA (Yu et al., 2017).

STAT3 is a transcription factor that modulates many genes related to apoptosis and EMT. STAT3 signaling is an important pathway which is frequently activated in many tumors (Sherry et al., 2010; Moroishi et al., 2015). Tan IIA treatment was found to inhibit STAT3 activation in bladder cancer cells and human gastric cancer cells (Huang SY. et al., 2017; Zhang Y. et al., 2018). This effect can inhibit the migration and invasion of hepatocellular carcinoma cells when combined with sorafenib/SC-1 (Chiu et al., 2018).

The hippo pathway is conservative signaling in mammals, and consists of MST1/2 (mammalian Sterile 20-like kinase 1/2) and LATS1/2 (large tumor suppressor 1/2) which could phosphorylate and inactivate the downstream transcriptional effector’s YAP/TAZ. The activation of the oncogene YAP or TAZ of the Hippo pathway results in liver tumorigenesis (Moroishi et al., 2015). Tan IIA can inhibit the transcriptional activity of YAP, a transcriptional effector at the downstream end of the hippo pathway, and thus inhibit the progress of cancer stem cells (Qin et al., 2018).

The transforming growth factor-β (TGF-β) signaling pathway is usually overexpressed in many disease states, such as fibrosis, inflammation and cancer, and the activation of TGF-β signaling promotes cancer cell migration and invasion. Smad7 is a negative regulator of the TGF-β signaling pathway and it can stably bind to the cytoplasmic domain of the activated type I receptor and block Smad2/3 phosphorylation (Sun et al., 2017). Cross-talk between TGF-β/SMAD and hippo/Yap signaling pathways was found to be crucial for tumorigenesis. Through experiments *in vitro*, and *in vivo,* qRT-PCR and WB assays, it found that Tan IIA upregulated SMAD7 to promote E3 ligase βTrcp expression resulting in promoting YAP protein degradation in liver cancer. Therefore, Tan IIA mediates the SMAD7-YAP expression in a TGF-β/SMAD signaling pathway-dependent manner to induce apoptosis and inhibit growth and migration in hepatocellular carcinoma cells (Ma et al., 2019).

In solid tumors, Tan-IIA inhibits the formation and metastasis of vascular endothelial cells by down-regulating the expression of hypoxia-inducible HIF-1α and inhibiting the secretion of the angiogenic factor under hypoxia (Zhou et al., 2020).

### Endoplasmic Reticulum Stress and Energy Supply

The endoplasmic reticulum stress (ERS), glycolysis and mitochondria dependent pathways are closely associated with cell apoptosis. Various ERS stimuli, such as oxidative stress, the accumulation of unfolded or misfolded proteins, and viral infection, can disturb cell homeostasis.

Tan IIA can decrease the expression of binding immunoglobulin protein (BIP), the main molecular chaperone in ER, and then activating C/EBP homologous protein (CHOP) which has been demonstrated to inhibit the protein expression of Bcl-2 (Zhang et al., 2017). Overexpression of Bcl-2 is often considered as a protective effect of various apoptotic stimuli, while down-regulation of Bcl-2 may induce apoptosis of tumor cells (Huang et al., 2020). Western blotting analysis indicated that Tan IIA activated the upstream elements, such as inositol-requiring enzyme (IRE) 1α and protein kinase RNA-like endoplasmic reticulum kinase (PERK), consequently resulted in an increase in their downstream targets eukaryotic initiation factor (eIF) 2α, *p*-JNK and CHOP in a dose-dependent manner in BxPC-3-derived xenograft tumors (Chiu and Su, 2017). These results indicated that Tan IIA induced ER stress *in vitro* and *in vivo*.

Mitochondria homeostasis can supply sufficient ATP to the cancer cell. Mitochondrial damage can initiate a caspase-9-related mitochondrial apoptotic pathway. Studies have identified mitochondrial homeostasis as a novel target for controlling tumor survival, migration, and proliferation. On the one hand, Tan IIA increased JNK phosphorylation and Mff expression in sw837 colorectal cancer cells, resulting in mitochondrial damage mediated by activation of mitochondrial fission *via* the JNK-Mff pathway (Jieensinue et al., 2018). On the other hand, After Tan IIA administration, mitochondrial fusion proteins Mfn1/2 and Opa1 were significantly decreased, while the fission protein Drp1 was significantly increased. So mitochondrial dysfunction was related to Tan IIA induced apoptosis and anti-angiogenesis in both osteosarcoma 143B cells and 143B cell xenograft mice (Huang ST. et al., 2017). Moreover, Tan IIA/IL-2 cotreatment amplified INF2-related mitochondrial fission via the Mst1-Hippo pathway (Qian et al., 2018), evoking cell death.

AMPK, an important kinase that regulates energy homeostasis, is one of the central regulators of cellular energy metabolism. Meanwhile, Nrf2 is associated with decreased oxidative stress. Investigations illustrated that the transcriptions, expressions, and activities of AMPK and Nrf2 were inhibited by Tan IIA, which significantly reduced survival, migration and invasion in MG63 osteosarcoma (Xie Z. et al., 2020). The functional assay showed that Tan IIA inhibited AMPK pathway and resulted in S-phase kinase associated protein 2 (Skp2) inactivation. The decreased Skp2 level failed to activate Parkin, resulting in the inhibition of mitophagy and enhancing colorectal cancer apoptosis (He and Gu, 2018).

The energy required for cancer cell proliferation can be obtained through glycolysis and is associated with the upregulation of key enzymes involved in glycolysis and glucose transporters. Tan IIA treatment inhibited glucose uptake and extracellular lactate production in SiHa cells. Glucose transporter 1 (GLUT1), an important glucose transporter, is a rate limiting enzyme in glucose transport. Pyruvate kinase M2(PKM2), a key and final enzyme in glycolysis, is highly expressed in tumor cells. GLUT1 and PKM2 were downregulated in response to Tan IIA treatment (Liu Z. et al., 2019). Further study on the microRNAs regulation of PKM2 showed that upregulated miR-122 expression could suppress PKM2 expression (Zhang et al., 2016). Thus, it leads to cancer cell apoptosis.

## Cardiovascular System

Tan IIA exhibits potent cardioprotective effects. Clinical studies have shown that STS has a good effect in the treatment of cardiovascular diseases, reduces the level of highly sensitive C-reactive protein and other circulating inflammation markers in patients with coronary artery disease (Li S. et al., 2017). In the animal or cell model of doxorubicin induced cardiotoxicity, Tan IIA pretreatment decreased the activity of myocardial enzymes, increased the activities of antioxidant enzymes superoxide dismutase, catalase and glutathione, and induced the nuclear accumulation of Nrf2 and its downstream genes in mice heart tissue and H9c2 cells. (Guo et al., 2018). In the model of H_2_O_2_ or DOX simulating oxidative stress, by regulating the miR-133a-3p/EGFR axis in H9c2 cells, the reduction of G0/G1 arrest induced by H_2_O_2_ was reversed (Xu H. et al., 2020). And it was found that Tan IIA could up-regulate miR-133 and inhibit caspase-9 signaling cascade, which improved myocardial apoptosis (Feng et al., 2016b; Song et al., 2017). Tan IIA can promote cardiac differentiation and improve cell motility by regulating Wnt/β-catenin signaling pathway (Li K. et al., 2020). Moreover, Tan IIA attenuates β-catenin and IGF-2R pathways and reduces subsequent apoptosis and remodeling while increasing survival proteins in AngII induced H9c2 cells (Chen et al., 2017).

**FIGURE 2:**
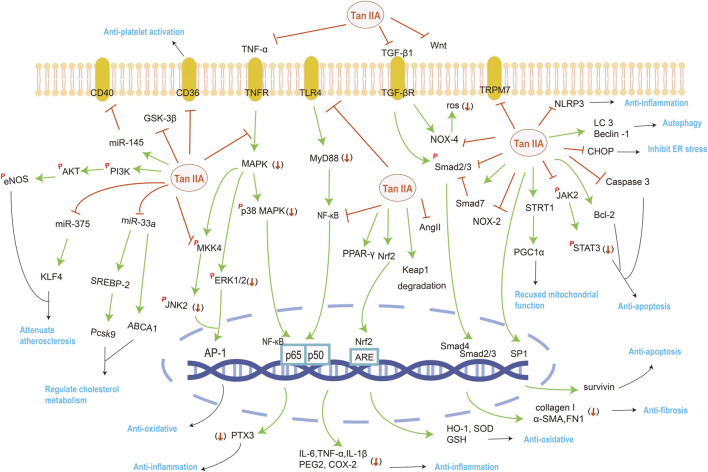
The mainly affected targets and pathways by Tan IIA against various system disorders. The orange red symbols (↓), ┤ and *p* represent down-regulation, inhibition and phosphorylation of proteins respectively, and green line-arrow “→” means singal transduction.

The mainly affected targets and pathways by Tan IIA against cardiovascular system diseases were summarized in Figure 2 and Table 2.

**TABLE 2:** Summary of the therapeutic effects and mechanisms of Tan IIA on cardiovascular system disorders.

Cardiovascular system disorders	Animal/cell	Mechanisms	Effect	References
Atherosclerotic	Apoe^−/−^ mice	↑TGF-β/PI3K/Akt/eNOS pathway	↓serum lipids, stabilize atherosclerotic plaques	Wang et al. (2020c)
		↓MMP-9, VEGF, and HIF1-α	↓endothelial injury, inflammatory damage	
Atherosclerotic	Apoe^−/−^ mice C57bl/6 mice	↓TLR4/MyD88/NF-κB Signal pathway	stabilize vulnerable AS plaque	Chen et al. (2019c)
Hyperlipidemia	Hyper lipidemia rats	↓ miR-33a	↓lipid deposition	Jia et al. (2016)
		↑ABCA1,SREBP-2, Pcsk9	↑histopathology in the rat liver tissue	
Atherosclerosis	Apoe^−/−^ mice	↓miR-375↑ KLF4	induced macrophages in atherosclerotic plaque to M2 type	Chen et al. (2019b)
Atherosclerosis	Apoe^–/–^ mice Raw264.7 cells	↓Bax and Cleaved-caspase-3 up-regulation	↓ox-LDL-induced apoptosis of VSMCs ↓ox-LDL-induced proliferation and migration of RAW264.7 cells	Wang et al. (2017a)
		↓levels of MMP-2, MMP-9		
		↓overexpression of TNF-a, IL-1β, IL-6, and MCP-1		
Angiotensin II-induced proliferation and autophagy	Vascular smooth muscle cells	↓MAPK signaling pathway	↓ Ang II-induced proliferation and autophagy of VSMCs	Lu et al. (2019)
Ages-induced proliferation and migration of VSMCs	Vascular smooth muscle cells	↓ERK1/2 MAPK signaling pathway	↓ AGEs-induced proliferation and migration of VSMCs	Lu et al. (2018)
Homocysteine-induced proliferation of vascular smooth muscle cells	Vascular smooth muscle cells	↑miR-145	↓ viability of VSMCs	Li et al. (2020b)
		↓CD40	↓VSMCs proliferation induced by Hcy	
Ischemia reperfusion injury	Sprague-Dawley rats cardiomyocytes from neonatal rats	↑PI3K, *p*-Akt/Akt, mTOR and *p*-eNOS/eNOS	↓apoptotic cells	Li et al. (2016b)
Hypoxia/reoxygenation	Myocardial microvascular endothelial cell	↓*p*-JAK2, *p*-STAT3, p53, Bax and Caspase-3	attenuate H/R-induced MMEC apoptosis	Cui et al. (2016)
Ischemia-reperfusion injury	C57bl/6 mice cardiac microvascular endothelial cells	↑SIRT1-PGC1α signaling pathway	↓CMEC apoptosis, preserving microvascular structure and function	Zhong et al. (2019)
Anoxia/reoxygenation injury	H9c2 cell	↑14-3-3η	↓cell apoptosis	Zhang et al. (2018c)
		↓cyt c,Caspase-3		
Ischemia re-perfusion injury	Mouse H9c2 cells	↑AK003290	alleviated H/R induced apoptosis, oxidative stress and loss of mitochondrial membrane potential	Chen et al. (2020a)
Pressure overload-induced heart failure	Sprague-Dawley rats	↓serum BNP	alleviating ventricular remodeling	Li et al. (2019b)
		↓ IL-6, serum CRP level, Bax protein	↓cardiomyocyte apoptosis	
Heart failure post-myocardial	Sprague-Dawley rats	↑ AMPK	↑cardiac function	Zhang et al. (2019a)
	H9c2 cell	↓ mTOR	↓ apoptosis, induce autophagy ↑ cellular viability	
Atrial fibrillation and chronic heart failure	Rabbits	↑ aERP	↓ inducibility of AF	He et al. (2016)
		↑interatrial conduction time		
		↑atrial post-repolarization refractoriness		
Cardiac fibrosis	C57bl/6 mice	↓NADPH oxidase2	↓ myocardial fibrosis	Huang et al. (2018)
Cardiac fibrosis	Neonatal rat cardiac fibroblasts	↓TGF-β1/Smad signaling	↓ high glucose-mediated collagen synthesis	Tsai et al. (2018)
Cardiac hypertrophy in spontaneously hypertensive	Spontaneously hypertensive rats	↑ *p*-eNOS、eNOS	protective effect on cardiac hypertrophy	Feng et al. (2016a)
	Wistar-Kyoto rats	↓Cys-C and Wnt expressions		

### Atherosclerosis

Atherosclerosis (AS) is the main cause of cardiovascular disease in the world. The underlying pathogenesis of AS involves endothelial dysfunction, imbalanced lipid metabolism and chronic inflammation caused by maladaptive immune response.

A clinical trial found that STS increased serum soluble potassium levels in patients on maintenance hemodialysis, and soluble potassium was able to decrease peroxide induced endothelial cell apoptosis and reduce the incidence of cardiovascular events in patients (Xu Q. et al., 2020). Injection of STS to atherosclerotic mice revealed a significant upregulation of eNOS phosphorylation and Akt phosphorylation, and contributed to the synthesis and release of endogenous nitric oxide by activating the TGF- β/PI3K/Akt/eNOS pathway, thereby serving as a component of the protection of endothelial cells (Wang et al., 2020c). Besides, studies shown that Tan IIA prevents endothelial inflammation by attenuating the expression of Pentraxin 3, a novel diagnostic biomarker for atherosclerosis, and Pentraxin 3 dependent monocyte adhesion to endothelial cells (Fang et al., 2018b).

Whether plaques are stable depends on the proportion of extracellular lipids, collagen, macrophages, or vascular smooth muscle cells (VSMCs). Tan IIA significantly lower serum lipid level, attenuate lipid deposition, and stabilize atherosclerotic plaque by anti-inflammatory and immunomodulatory damage repair effects (Wang et al., 2020c).

On the one hand, Tan IIA inhibits miR-33a overexpression and modulates SREBP-2/Pcsk9 signaling pathway proteins to regulate cholesterol metabolism in hyperlipidemic rats (Jia et al., 2016). A previous study found that an intronic microRNA, miR-33, located within the *SREBF2* gene, suppressed the expression of the cholesterol transporter ABC transporter A1 (ABCA1) and reduced HDL levels. Conversely, inhibition of miR-33 increased ABCA1 and circulating HDL levels (Rayner et al., 2011). Tan IIA treatment attenuated lipid deposition in the livers of hyperlipidemic rats and suppressed the expression of miR-33a, upregulated the protein expression levels of ABCA1, SREBP-2 and Pcsk9. These suggest that miR-33 antagonism may be atheroprotective. On the other hand, Tan IIA downregulates the key inflammation and immunity protein expression in the TLR4/MyD88/NF-κB pathway in ApoE^−/−^ mice (Chen Z. et al., 2019). Tan IIA could inhibit miR-375 then activate KLF4 to induce macrophages in atherosclerotic plaque to M2 type while attenuating polarization of M1 type to attenuate atherosclerosis in mice and ox-LDL induced Raw264.7 cells (Chen W. et al., 2019).

In addition to inhibiting macrophage migration, Tan IIA also inhibits apoptosis, hyperproliferation, and autophagy in VSMCs (Lu et al., 2019). Tan IIA not only has obvious anti-inflammatory properties, but also inhibits ERK1/2 MAPK signal transduction, thus inhibiting VSMCs cell proliferation and migration induced by advanced glycation end products (Wang B. et al., 2017; Lu et al., 2018). miR-145 can effectively restrain the proliferation of VSMCs. Tan IIA up-regulates the expression of miR-145 and down-regulates its target gene *CD40* to inhibit VSMCs proliferation (Li et al., 2020b). The combination of Tan IIA and astragaloside IV may stabilize vulnerable plaques in the ApoE−/− mice, visibly reducing the cytoplasmic lipid droplet accumulation induced by ox-LDL (Wang N. et al., 2020). For the treatment of atherothrombosis, platelet activation studies *in vitro* demonstrated that Tan IIA prevented platelet-derived microvesicles induced platelet activation by down-regulating CD36 and MKK4/JNK2 signaling pathway (Wang H. et al., 2020). It is worth mentioning that the exploration of the combination of experimental design and computer technology is more and more popular. Bioinformatics result showed that the most significant pathways regulated by Tan IIA were associated with inflammation, and involved in the signaling pathways of Ras, Rap1, MAPK, cAMP, T cell receptor, and so on (Chen W. et al., 2020).

### Ischemia/Reperfusion (IR)injury

Myocardial ischemia refers to the myocardial tissue without blood flow, resulting in oxygen supply and demand imbalance, eventually leading to tissue dysfunction or injury, known as myocardial ischemia-reperfusion injury (MIRI). The pathogenic factors of MIRI include reactive oxygen species (ROS), endothelial dysfunction and inflammation.

Tan IIA enhanced the expression of eNOS and mTOR through the activation of the PI3K/AKT pathway, thereby promoting the production of endogenous NO to relieve MIRI in rats (Li Q. et al., 2016). Inhibiting the expression of *p*-JAK2, *p*-STAT3, p53, Bax, Caspase-3 and Bcl-2 can reduce myocardial apoptosis (Cui et al., 2016). Pretreatment with Tan IIA sustaining the mitochondrial functions, it may associate with upregulation of 14-3-3η and SIRT1/PGC1α pathway activation. Upregulation of 14-3-3η promotes Bcl-2 translocation to the outer mitochondrial membrane, prevents mitochondrial permeability transition pore opening, reduces cytochrome c release, prevents Caspase-3 activation, and inhibits cardiomyocyte’s apoptosis. The activation of SIRT1/PGC1α pathway maintains mitochondrial energy metabolism, which is beneficial to keep mitochondrial function, cardiac microvascular endothelial cell survival and microvascular homeostasis (Zhang et al., 2018c; Zhong et al., 2019).

Moreover, bioinformatic tools exert more effect on the predicted target genes. It was reported that bioinformatics tools predicted lncRNA AK003290 as a potential target of miR-124-5p, and experiments further confirmed that miR-124-5p directly targeted AK003290. According to the qPCR results, AK003290 was found to be downregulated by hypoxia/reoxygenation treatment, while Tan IIA up-regulated the expression of AK003290. Both AK003290 knock down and miR-124-5p overexpression can reverse the effect of Tan IIA (Chen L. et al., 2020).

### Heart Failure (HF)

Heart failure is a syndrome that causes circulatory dysfunction due to absolute or relatively insufficient cardiac output when the venous blood flow is sufficient. Heart failure is the final stage of heart disease development and may be accompanied by ventricular remodeling, arrhythmia, autophagy, myocardial fibrosis, and cardiac hypertrophy.

After establishing a pressure overload heart failure model in rats by abdominal aortic constriction, the left ventricular ejection fraction (LVEF), left ventricular fractional shortening (LVFS) and the left ventricular end diastolic diameter (LVIDd) were significantly increased, and the left ventricular end systolic diameters (LVIDs) were significantly decreased in the Tan IIA treated group. Its changes may be associated with attenuating the inflammatory response and cardiomyocyte apoptosis (Li X. et al., 2019).

In the chronic heart failure whole heart model, Tan IIA had no effect on the atrial action potential duration (AAPD) time course but increased the refractory period (AARP), resulting in a significant increase in the refractory period after atrial repolarization. Therefore, it can effectively reduce atrial fibrillation inducibility, and the main reasons for antiarrhythmic are prolongation of the atrial repolarization refractory period and a moderate increase in atrial conduction time (He et al., 2016).

Tan IIA can up-regulate the expression of LC3 and Beclin1 and inhibit the expression of p62 in HF model induced by ligation of left anterior descending branch and H9c2 cell injury model induced by H_2_O_2_, Further experiments suggesting that Tan IIA can enhance autophagy and inhibit apoptosis via activation of the AMPK-mTOR signaling pathway (Zhang et al., 2019a).

The anti-fibrosis effect of Tan IIA is partly related to the reduction of ROS production by inhibiting NADPH oxidase 2, partly by reducing the expression of TGF-β1and inhibiting TGF-β1–Smad2/3 signal transduction. The NOX family proteins are enzymes dedicated to the generation of O^2−^ and/or H_2_O_2_ and NOX2 may mediate LPS-induced cardiac fibrosis. TGF-β1 is a key regulator of cell proliferation, differentiation, migration, immune regulation and extracellular matrix (ECM) transformation in fibrotic diseases. In silicosis fibroblasts, it was observed that TGF-β1 induced the expression of *p*-Smad2 and *p*-Smad3, downregulated the expression of Smad7 in a dose-dependent manner. Consequently, the left ventricular collagen fraction area and the activation of fibrosis related genes were reduced, and the proliferation and collagen synthesis of cardiac fibroblasts were inhibited (Huang et al., 2018; Tsai et al., 2018).

Activation of Wnt signaling pathway leads to hypertrophy and growth of cardiomyocytes, Cys-C plays a promoting role in cardiac hypertrophy. Tan IIA had a positive effect on cardiac hypertrophy by reducing Cys-C and Wnt expression, and it decreased the extent of cardiomyocyte swelling and the area of individual cardiomyocytes in the treatment group (Feng et al., 2016a). The combination of Tan IIA and puerarin exhibited favorable effects on improving hemodynamics and immersion of inflammatory cells (Gao et al., 2019). In conclusion, Tan IIA can improve hemodynamic and electrophysiological parameters, has a protective effect on myocardial fibrosis and hypertrophy and slow down the progress of heart failure.

## Nervous System

Literatures have reported the effects of Tan IIA on Alzheimer’s disease (AD) and neuroprotective. The mainly affected targets and pathways by Tan IIA against nervous system diseases were summarized in Figure 2 and Table 3.

**TABLE 3:** Summary of the therapeutic effects and mechanisms of Tan IIA on nervous system disorders.

Nervous system disorders	Animal/Cell	Mechanisms	Effects	References
Alzheimer’s disease	SH-SY5Y Cells	↓COX-2 expression and PGE2 synthesis	↓Aβ-mediated cell viability reduction, apoptosis induction and pro-inflammatory effect	Geng et al. (2019)
Alzheimer’s disease	APP/PS1mice Wild-Type mice	↓CHOP and *p*-JNK	↓deposition of Aβ plaques and neuronal apoptosis	He et al. (2020)
		↓caspase-3 activity		
		↑the ratio of Bcl-2/Bax		
Alzheimer’s disease	SH-SY5Y cells	↓GRP78, eIF2α and ATF6, cytochrome c, cleaved caspase-9 and cleaved caspase-3, the activity of caspase-3/7	↓apoptosis	Yang et al. (2019)
		↑the ratio of Bcl-2/Bax, MMP and ATP content	↑cell viability	
Alzheimer’s disease	Sprague-Dawley rats	↓ ERK and GSK-3β activity	↓Tau hyperphosphorylation	Lin et al. (2019)
Alzheimer’s disease	APP/PS1 mice Wild-Type mice BV2 Cells	↓mRNA levels of TNF-α, IL-6, and IL-1β	↓Aβ plaques,microglial and astrocytic activation,spatial learning and memory deficits	Ding et al. (2020)
	U87 Cells	↓RAGE and the *p*-IκBα and NF-κB p65		
Alzheimer’s disease	Swiss Albino Mice	↑the SOD and GSH-Px activities ↓the MDA level restoring the AChE activity	↑cognitive impairment,ameliorating neuronal damage, restoring cholinergic function, attenuating oxidative stress	Liu et al. (2016)
Cognitive dysfunction	Sprague-Dawley rats	↓caspase-3, caspase-8, and caspase-9,level of MDA	alleviated learning memory and cognitive dysfunction	Liao et al. (2020)
		↑activity of SOD and GSH-Px		
Alzheimer’s disease	U87 cells	↓TLR4/NF-κB/MAPKs、IL-1β, TNF-α, and IL-6	↓LPS-induced neurotoxicity and neuroinflammation	Jin et al. (2020)
Cerebral ischemia	HT-22 cells	↑PI3K/Akt/mTOR	↓autophagy、cell death	Zhu et al. (2017)
		↓ROS		
Ischemia-reperfusion injuries	SH-SY5Y cell	↑miRNA-135b,AMPK	↓OGDR-induced viability reduction and apoptosis	Weng et al. (2018)
Hypoxic ischemic encephalopathy	C57BL/6J mice Experimental mice neurons	↓TLR-4、NF-κB	↓neuronal apoptosis、infarct volume and neuronal degeneration in mice	Fang et al. (2018a)
Cerebral ischemia reperfusion	GLUT1 knockdown mice	↑PI3K/mTOR/HER3	↑viability of neurons and the recovery of brain function	Wang et al. (2020d)
	GLUT1 overexpression mice			
	C57BL mice			
	Neuro-2a cells			
Cerebral ischemia	Sprague–Dawley rats	↑ATPase	↓deficits in energy metabolism cell death	Wen et al. (2016)
		↓microglial activation		
Cerebral ischemia/reperfusion	Nrf2 knockout mice	↑Nrf2 mRNA and the contents of Nrf2 protein	↓generation of oxidative productions	Cai et al. (2017)
			↑contents of antioxidant enzymes	
Cerebral ischemia	PC-12 Cells	↓ miR-28	↓hypoxia induced PC-12 cell injury	Tang et al. (2019)
		↑Sp1/survivin		
Cerebral ischemia	Hippocampal neurons from E18 embryonic rats	↑ UQCRFS1 expression	↓cells apoptosis and mitochondria dysfunction	Dai et al. (2017)
Glutamate-mediated toxicity	SH-SY5Y cells	↑ Bcl-2 protein level	↓glutamate-induced apoptosis prevents glutamate-induced mitochondrial dysfunction	Li et al. (2017a)
		↓ Bax and cleaved caspase-3 levels		
		↓JNK and p38 MAPK activation		
Diabetic neuropathic pain	Sprague-Dawley rats	↑Nrf2/ARE signaling pathway ↓NF-kB signaling pathway	alleviates neuropathic pain	Feng and Qiu, (2018)

### Alzheimer’s Disease (AD)

Intravenous injection of STS reduced the leakage and injury of the blood-brain barrier and improve the neurological prognosis of patients with acute ischemic stroke after recombinant tissue plasminogen activator treatment (Ji et al., 2017). Morris water maze test showed that Tan IIA significantly ameliorated the spatial learning and memory impairment of rats, and improved the cognitive impairment of AD model. The underlying mechanisms are related to reducing Tau protein phosphorylation, attenuating the neurotoxicity of amyloid beta (Aβ), anti-inflammatory and antioxidant stress, and protecting neurons.

Gsk-3β is a key kinase that plays an important role in AD-like Tau hyperphosphorylation. ERK and JNK (MAPK family) activation can also phosphorylate Tau protein at ser396 and ser404 sites. Tan IIA can reduce the expression of Tau and attenuate Tau phosphorylation in cells through downregulation of the activity of ERK and GSK-3β (Lin et al., 2019) and induce Tau degradation. Molecular docking and molecular dynamic study predicted that Tan IIA could strongly bind to the Tau binding site (Cai et al., 2020).

In addition, Tan IIA decreased Aβ plaques deposition in the parietal cortex and hippocampus (He et al., 2020), ameliorated Aβ-induced neurotoxicity by downregulating COX-2 expression and PGE2 synthesis (Geng et al., 2019), and suppressing ER stress via suppression of CHOP and JNK pathways (Yang et al., 2019).

### Neuroprotection

The anti-oxidation effect of Tan IIA is reflected in the up-regulation of Nrf2 mRNA expression, the increase of antioxidant enzyme content and the decrease of oxidation products (Cai et al., 2017). For example, Tan IIA increased the activities of SOD and GSHP_X_, and reduced the formation of ROS *via* decreasing the malondialdehyde (MDA) level and improving the cholinergic system *via* restoring the AChE activity (Liu et al., 2016; Liao et al., 2020).

Studies found that Tan IIA has obvious anti-inflammatory activity *in vitro* and *in vivo* (Ding et al., 2020). Pretreatment with Tan IIA can significantly promote neutrophil apoptosis (Gong et al., 2020b) and regulate the TLR4-mediated NF-κB/MAPKs signaling pathways, obviously inhibit the generation of pro-inflammatory cytokines and mRNA transcription, thereby reduce the damage of nerve cells caused by ischemia and hypoxia (Fang C. et al., 2018; Jin et al., 2020). Further studies found that the upregulation of miRNA-135b by Tan IIA downregulates AMPK phosphatase Ppm1e. Knockout of Ppm1e or forcing miRNA-135b expression also activates AMPK and protects SH-SY5Y neuronal cells from oxygen-glucose deprivation and re-oxygenation damage (Weng et al., 2018).

Besides, Tan IIA inhibits hypoxia induced up-regulation of miR-28 and activates SP1/survivin pathway to reduce cell apoptosis (Tang et al., 2019), increase ATPase, maintain the activity of Na^+^/K^+^-ATPase (Wen et al., 2016; Zhu et al., 2017), save the loss of MMP and the decrease of ATP content, and increase the expression of Rieske iron-sulfur polypeptide 1 to help maintain and restore mitochondrial function (Li H. et al., 2017; Dai et al., 2017). Additionally, Tan IIA enhances glucose uptake to promote the recovery of brain function (Wang et al., 2020d). By evaluating motor behavior and tibialis anterior muscle group, and histological analysis of sciatic nerve and lumbar spinal cord, Tan IIA can reduce the injury and promote the regeneration of sciatic nerve in rats (Li M. et al., 2017; Wang et al., 2018). Diabetic chronic hyperglycemia and its pathophysiological changes lead to nervous system damage and pain. It is one of the most common, complex and serious complications in diabetic patients. Behavioral tests showed that Tan IIA had protective effects on the sciatic nerve, reduced diabetic neuropathic pain, and exerted significant antiallodynic and anti-hyperalgesic effects in experimental rats by suppressing inflammation (Zhang B. et al., 2018; Feng and Qiu, 2018). Patch clamp recordings revealed that in the diabetic rats, Tan IIA treatment effectively restored a subnormal state of increased excitability of dorsal root ganglion (DRG) nociceptive neurons by preventing increases in both Tetrodotoxin-resistant (TTX-resistant) and Tetrodotoxin-sensitive (TTX-S) sodium currents. Furthermore, protein expression of voltage gated sodium channel (VGSCs) α- Subunits Nav1.3, Nav1.7 and Nav1.9 increased in DRG and normalized by Tan IIA (Ri-Ge-le et al., 2018). Other literature reported that Tan IIA could promote Treg cell differentiation (Gong et al., 2020a) and improve depression like behavior in mice (Lu et al., 2020).

## Respiratory System

Tan IIA has therapeutic effects on respiratory diseases, especially acute lung injury with pulmonary fibrosis. The mainly affected targets and pathways by Tan IIA against respiratory system diseases were summarized in Figure 2.

### Acute Lung Injury (ALI)

ALI is a clinical disease in which pulmonary capillary endothelial cells and alveolar epithelial cells are damaged, followed by pulmonary edema, leading to hypoxemia. Tan IIA had an obvious ameliorative effect on alveolar structure destruction and exudative edema in mice with LPS induced ALI by reducing inflammatory factors. It is possible that Tan IIA could enhance the expression of Sirt1, thereby promoting cellular p65 protein deacetylation, inhibiting NF-kB transcriptional activation, then inhibit the NF-kB mediated inflammatory process (Quan et al., 2019). Amelioration of acute lung injury by Tan IIA can also inhibit proinflammatory factors by inhibiting the expression of TRPM7 and reducing calcium influx in lung interstitial macrophages (Li J. et al., 2018), as well as preventing nucleotide-binding oligomerization domain (NOD)-like receptor family protein 3 (NLRP3) inflammasome activation (Chen T. et al., 2019).

### Silicosis

Silicosis is caused by long-term exposure to free crystalline silica (SiO_2_) particles, disease progression includes persistent pulmonary inflammation and excessive production of extracellular matrix, which eventually leads to irreversible destruction of normal lung structure and pulmonary fibrosis. At present, there is no effective drug treatment (Lian et al., 2017). It is recognized that TGF-β1-Smad signaling axis is the main way to induce pulmonary fibrosis. In a silicosis rat model, Tan IIA significantly relieved silica-induced lung fibrosis by histological and immunohistochemical analyses, ameliorate destructive pathological alterations and collagen deposition, and downregulate the expression of the ECM proteins collagen I, α-SMA, and FN1 in rats. Tan IIA attenuates silica induced pulmonary fibrosis is associated with Nrf2, NOX4 and TGF-β1/Smad signaling pathway. Tan IIA treatment effectively inhibited TGF-β1-induced phosphorylation of Smads, especially the continuous phosphorylation of Smad3 in the nucleus, and up-regulated the expression of Smad7 in silico cells, resulting in decreased ECM deposition (Feng et al., 2020b). Nrf2 is a positive regulator of antioxidant enzymes and genes. Tan IIA increased the induction of Nrf2 by promoting the degradation of Keap1 and weakening the binding of Keap1 to Nrf2. By activating Nrf2, Tan IIA reduced the availability of glutamate in the tricarboxylic acid cycle by transferring glutamine hydrolysis to GSH production. Thus, Tan IIA activated Nrf2/GSH signaling pathway to limit glutaminolysis in myofibroblast proliferation (An et al., 2019). Besides, Nrf2 knockdown by siRNA partly blocked the effects of Tan IIA on EMT and TGF-β1/Smad signaling activation induced by silica. So Tan IIA may attenuate silica-induced pulmonary fibrosis via Nrf2-mediated inhibition of EMT and TGF-β1/Smad signaling (Feng et al., 2020a).

Additionally, TGF-β1 promotes ROS formation mainly by inducing the expression and activity of NOX4 in many cell types, while Tan IIA reduced NOX4 upregulated mRNA and protein levels in silico rats (Feng et al., 2019). Therefore, the anti-fibrosis effect of Tan IIA can also be attributed to the reduction of oxidative stress. Moreover, it is reasonable to think that the antioxidant potential of Tan IIA is associated with its ability to alter the composition of the AP-1 heterodimer. AP-1 is composed of Jun and Fos proteins that combine to form a functional dimer and are activated through MAPKs (JNK, ERK, and p38), which are signaling under hypoxia. Tan IIA activated JNK and ERK pathway that induced c-jun/c-fos, c-jun/fosB, junD/c-fos, and junD/fosB heterodimers to increase cells survival. This in turn leads to cell cycle progression through activation of cyclins (D and B), as further confirmed by lower levels of p53 and its downstream genes (p16, p21 and p27) (Yadav et al., 2020). Taken together, Tan IIA might be a potential therapeutic remedy for pulmonary fibrosis and injury repair.

## Urinary System

Tan IIA can attenuate ischemia/reperfusion induced kidney injury (Xu et al., 2016), renal fibrosis, as well as renal injury from various other causes. Administration of Tan IIA significantly reduced renal dysfunction on days 7 and 14 after experiencing folate induced acute kidney injury in mice. In addition, it markedly reduced the tubuloid interstitial accumulation of fibronectin and collagen I (Jiang et al., 2016) and reversed the HG-induced increase in α-SMA and decrease in E-cadherin, thereby reducing renal proximal tubular fibrosis (Cao et al., 2017). Tan IIA treatment increased immune cell precipitation in renal cells and improved the lipid and glucose metabolism, insulin resistance, and body weight in type 2 DM Rats (Yuan et al., 2018), attenuates renal injury by reducing excessive oxidative stress and inflammation, and the mechanisms include activating Nrf2, and upregulating heme oxygenase-1 (HO-1) expression (Liang et al., 2018), and enhancement of glutathione mediated detoxification pathways (Li W. et al., 2019). Summarily, the affected targets by Tan IIA against urinary system diseases were shown in Figure 2.

## Digestive System

Tan IIA treatment can promote the repair and regeneration of damaged liver (Ze et al., 2016). It is used to treat liver cirrhosis, nonalcoholic fatty liver disease and rifampicin induced cell damage.

Tan IIA could alleviate ECM accumulation, attenuate the proliferation and activation of hepatic stellate cells, and effectively improve liver fibrosis. Network pharmacology-based predictions found that the possible molecular mechanisms include MAPK, Wnt, PI3K/Akt signaling pathways through inhibition of c-Jun, p-c-Jun, c-Myc, CCND1, MMP9, P65, P–P65, PI3K and P38, which are validated by *in vitro* and *in vivo* experiments (Shi MJ. et al., 2020). Another study also confirmed that Tan IIA inhibiting fibrosis and reducing inflammation and oxidative stress via HO-1, Akt, and p38 MAPK signaling pathways in a rat model of cirrhosis (Shu et al., 2016).

In studies of nonalcoholic fatty liver disease (NAFLD), Tan IIA was found to improve NAFLD by targeting PPAR-γ and TLR4, thereby reducing lipids and oxidative stress, a strategy that may form the basis for novel NAFLD therapies (Huang et al., 2019).

Rifampicin (RFP)—induced biliary homeostatic liver injury is characterized by impaired hepatic bile acid (BA) transport. Bile salt efflux pump (BSEP) and Na ^+^/taurocholate cotransporter (NTCP) are the main transporters of BA. The mRNA expression of NRF2, BSEP and NTCP was strongly induced by Tan IIA combined with RFP. Nrf2 plays an important role in directly activating BSEP and NTCP expression, The expression of epigenetic modification-related proteins in terms of DNA methylation was investigated and it found that Tan IIA activated Nrf2 through ten-eleven translocation two demethylations at specific CpG sites. And Nrf2 knockout mice were more susceptible to RFP induced liver injury, while BA transporters on Nrf2 signaling pathway were changed to some extent. This suggests that NRF2 activation by Tan IIA may favor RFP induced cholestatic liver injury (Yang et al., 2020). Summarily, the affected targets by Tan IIA against digestive system diseases were shown in Figure 2.

## Motor System

Tan IIA has been used to treat arthritis as well as post fracture healing (Wang Y. et al., 2019), mainly through anti-inflammatory effects. Fibroblast-like synoviocytes from patients with RA are termed RAFLS, the major component of synovial tissues associated with joint damage. Dysregulated RAFLS proliferation is responsible for synovial hyperplasia and proinflammatory cytokine production, which exacerbates joint destruction. Tan IIA promotes fibroblast-like synoviocytes in rheumatoid arthritis apoptosis by upregulating lncRNA GAS5. (Li G. et al., 2018). In addition, Tan IIA reduces inflammatory injury of cells by downregulating miR-203a and inhibiting JAK/STAT and JNK pathways (Luan and Liang, 2018), and ameliorates the severity of arthritis in AIA mice (Du et al., 2020). From the perspective of metabolism and redox regulation, Tan IIA reduces HIF-1α induction by inactivation of succinate dehydrogenase, and preserves Sirt2 activity by downregulating glycolysis. It is helpful to inhibit the activation of NLRP3 inflammasome (Liu et al., 2021).

In the diabetic mouse model, Tan IIA was found to reduce the level of AngII *in vivo* circulation and bone by potentially targeting renin, thus improving the bone mineral density and microstructure of proximal tibia and increasing the trabecular bone area of the distal femoral end. It is beneficial for Tan IIA in the treatment of diabetic osteoporosis and the drug development of renin inhibitors (Zhang et al., 2020). Summarily, the mainly affected targets and pathways by Tan IIA against motor system diseases were shown in Figure 2.

## Conclusion and Perspectives

*S. miltiorrhiza* contains many phenolic acids and tanshinones, which are considered as the main ingredients responsible for its pharmacological effects. Tan IIA, as the most important one of the active components in *S. miltiorrhiza*, has been studied on the prevention and treatment of cardiovascular diseases in China. In zebrafish embryo at high concentrations, Tan IIA shows potential developmental deformity and cardiac toxicity (Wang T. et al., 2017). Tan IIA as a fat-soluble compound has poor oral bioavailability and could mainly stay in gastrointestinal tissue and not easily pass through biological barriers to arrive in brain and testes tissues. Tan IIA could be hydroxylated by CYP2A6 in liver microsomes, followed by glucuronidation and excreted via the bile. Tan IIA can induce the expression of the *CYP3A4* gene so that it should be prudent to take drugs metabolized by CYP3A4 when co-using *S. miltiorrhiza* products.

Meanwhile, more importantly, the present paper reviews the recent investigation progress during the years 2015–2021 on Tan IIA’s multiple pharmacological effects and mechanisms involving in multiple signaling molecules and multiple pathways (Figures 1, 2).

Tan IIA exerts anticancer activities mainly *via* inhibiting cancer cell proliferation, activating cancer cell apoptosis and autophagy and inducing cell cycle arrest, and restrains cancer invasion, migration and metastasis. The mechanisms refer to that Tan IIA can reduce the expression of EGFR, IGF1R, Her2, VEGFR, survivin, Bcl-2, Nrf2, miR30b, SLC7A11, upregulate PERK, ATF6, IRE1α, CHOP, PARP, caspase-3, caspase-8, caspase-9, caspase-12, Beclin-1, LC3-II, cyclin B1/CDC2, SHP2, *p*-JNK, Mff, Drp1, p53 and miR-205, and block the AMPK/Skp2/Parkin and PI3K/AKT/mTOR pathways to inhibit cancer cell proliferation and induce cancer cell apoptosis, autophagy and/or ferroptosis. Moreover, Tan IIA can decrease the expression of MMP-2, MMP-9, FOXM1 and HIF-1α, inhibit STAT3 activation and the transcriptional activity of YAP, and block the NF- kB, STAT3, Hippo, TGF-β signaling pathways to prevent the metastasis of tumor. In addition, Tan IIA could inhibit glucose uptake and extracellular lactate production, which could be manifested in the downregulation of GLUT1 and PKM2 in cancer cells, and ultimately results in cancer cell apoptosis.

Tan IIA can treat a variety of systematic diseases, including cardiovascular, nervous, respiratory, urinary, digestive, and motor systems disorders. The involved targets genes and proteins include miR-28, miR-33a, miR-135b, miR-145, miR-203a, miR-375, lncRNA GAS5, p16, p21, p27, p38, p53, p62, and ABCA1, ABCG1, SREBP-2, Pcsk9, KLF4, ERK1/2, CD40, CD36, Ras, Rap1, MAPK, 14-3-3η, cytochrome c, Bax, Caspase-3, LC3B, Beclin1, NADPH oxidase 2, TGF–β 1, Cys-C, Wnt, ERK, GSK-3β, COX-2, PGE2, SOD, GSHP_X_, Sirt1, TRPM7, NLRP3, α-SMA, MMP-2, MMP-9, TNF-a, IL-1β, IL-6, and MCP-1, FN1, Nrf2, NOX4, Nrf2, HO-1, CCND1, MMP9, PI3K, PPAR, TLR4 and SDH. Therefore, Tan IIA shows its many favorable activities in therapeutic effects containing anti-inflammatory, anti-oxidant stress, anti-apoptosis, anti-fibrosis, anti-ischemia/reperfusion injury, repairing immunomodulatory damage, promoting autophagy, restoring mitochondrial function, enhancing glucose uptake, *etc*., being mainly attributed to the multiple pathway regulation of Tan IIA on the Wnt/β-catenin, IGF-2R, SREBP-2/Pcsk9, NF-κB, MAPK, PI3K/AKT, SIRT1/PGC1α, TGF-β1/Smad, CHOP, JNK, Nrf2 and/or JAK/STAT signaling pathways.

**FIGURE 3:**
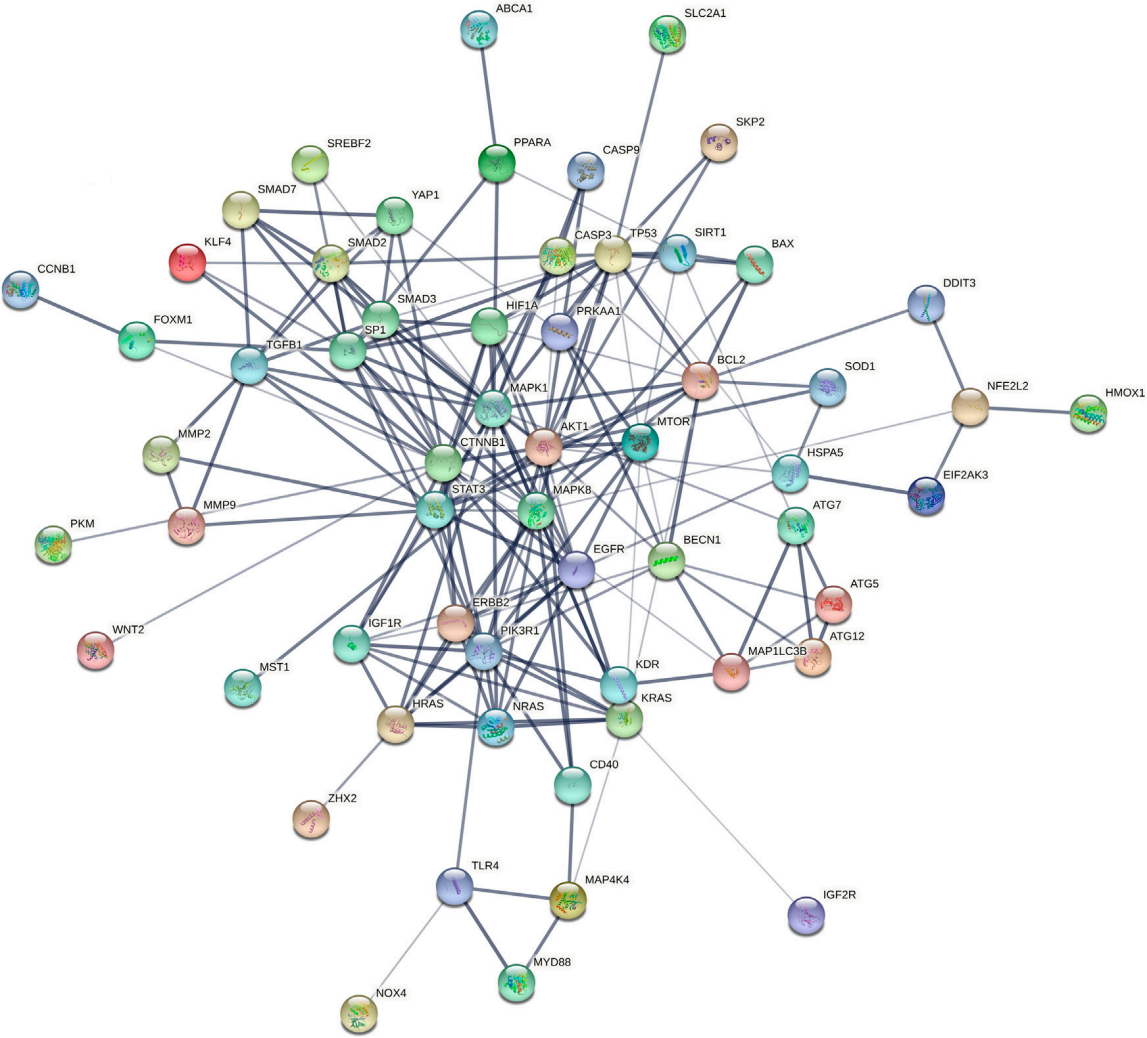
PPI network using the reported targets for Tan IIA (2015–2021). Network nodes (color balls with 3D protein structure known or predicted) represent target proteins. Edges (lines) represent protein-protein associations and line thickness indicates the strength of data support (the thicker the line, the greater the strength).

**FIGURE 4:**
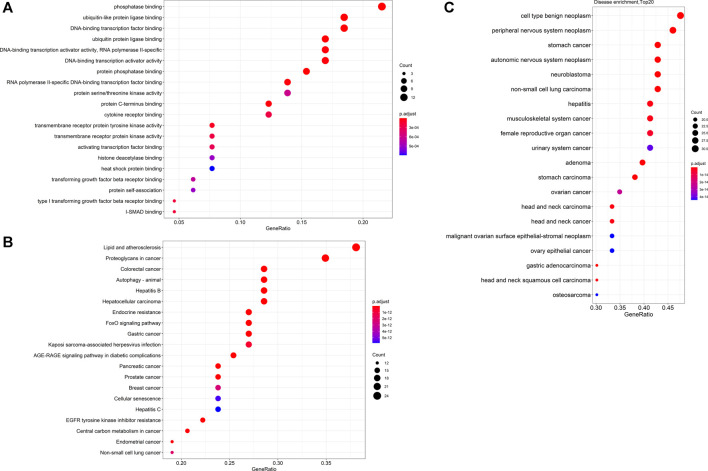
Dotplots of GO analysis **(A)**, KEGG analysis **(B)** and DO enrichment analysis **(C)** of proteins or genes previously reported in the literature.

Furthermore, the collection of targets genes and proteins abovementioned for Tan IIA is without doubt a good dataset to in depth predict the potential effects and underlying mechanisms of the active ingredient on diseases by bioinformatics analysis. To explore the possible relationship of targets abovementioned for Tan IIA, the potential protein interaction (PPI) network was contructed according to our previous report (Xie R. et al., 2020) by input the reported targets into the online STRING webserver (https://string-db.org). As a result, 65 nodes and 191 edges were generated in the PPI network afer hiding disconnected nodes. Among the target nodes, MAPK1 (degree: 20), AKT1 (degree: 20), and CTTNB1 (degree: 20), MAPK8 (degree: 17), and PIK3R1 (degree: 16) locate in the center of the PPI diagram (Figure 3) and were predicted as the most important roles in PPI network due to their high connective numbers (degree) with the other targets. Subsequently, GO, KEGG as well as DO (disease ontology) enrichment analyses of the reported targets were futher performed by using R package according to previous reports (Shi et al., 2020; Lin et al., 2021). Go is used to state various attributes of genes and gene products, and the analysis, as shown in Figure 4A, mainly involves phosphatase binding, ubiquitin-like protein ligase binding, DNA-binding transcription factor binding, ubiquitin protein ligase binding, DNA-binding transcription activator activity, RNA polymerase II-specific. KEGG is an information network connecting known intermolecular interactions, such as metabolic pathways, complexes, biochemical reactions, etc. As shown in Figure 4B, the mainly predicted pathways include Lipid and atherosclerosis, Proteoglycans in cancer, Colorectal cancer, Autophagy–animal, and Hepatitis B. Do is an analysis method that has an important role in the understanding of disease pathogenesis based on genetic studies of similar relationships to disease. The main genes were enriched in cell type benign neoplasm and peripheral nervous system neoplasm. It is also closely related to peripheral nervous system neoplasm, stomach cancer, autonomic nervous system neoplasm, neuroblastoma, etc.(Figure 4C)

According to the results of previous reports herein and the present GO, KEGG pathway and DO enrichment analyses, we provide the following ideas.

1) Firstly, the GO analysis results indicate that Tan IIA could mainly affect the phosphatase binding, ubiquitin-like protein ligase binding, and ubiquitin protein ligase binding, suggesting that Tan IIA could regulate the phosphorylation and ubiquitination of certain key proteins in cell biological processes. In a word, the regulation of Tan IIA on protein phosphorylation and ubiquitination could be pivotal mechanisms of this ingredient against diseases. Therefore, the roles of Tan IIA and these effects must be validated by *in vitro* and *in vivo* experiments with comprehensive and high throughput experimental technologies, such as phosphorylation and ubiquitination proteomic analyses.
2) Secondly, the current KEGG analysis results demonstrate that Tan IIA could affect the pathways including Lipid and atherosclerosis, Proteoglycans in cancer, Colorectal cancer, Autophagy, and Hepatitis B, which have been rarely reported on the Tan IIA’s effect mechanisms. It may be important to validate these predicted pathways by experimental evidence, such as western blot and quantitative reverse transcription PCR results for us to understand in-depth the therapeutic effects of the active ingredient.
3) Thirdly, the results of DO enrichment analysis show that Tan IIA could possess broad spectrum antitumor activity, mainly referring to cell type benignneoplasm and peripheral nervous system neoplasm. Although the literature has reported the extensive anticancer property, it slightly mentions the effect of Tan IIA on peripheral nervous system neoplasm, including malignant schwannoma, ganglioneuroma, pigmented malignant schwannoma, plexiform neurofibroma, *etc.* Therefore, maybe Tan IIA is a promising anti-peripheral nervous system neoplasm candidate, which requires further validation by *in vitro* and *in vivo* experiments for its drug development.
4) Ultimately, up to date, the real receptor targets of Tan IIA are still unclear. Target fishing for Tan IIA should be performed to extensively screen the possible target proteins or genes, and the results should then be further investigated by gene editing and compound-target complex crystallization experiments to disclose the real targets of this ingredient.

In conclusion, these ideas may provide new clues or perspectives to further investigate the therapeutic effects and mechanisms of Tan IIA, to promote the drug development and clinical applications of this ingredient.
